# Inhibition of FAO in AML co-cultured with BM adipocytes: mechanisms of survival and chemosensitization to cytarabine

**DOI:** 10.1038/s41598-018-35198-6

**Published:** 2018-11-15

**Authors:** Yoko Tabe, Kaori Saitoh, Haeun Yang, Kazumasa Sekihara, Kotoko Yamatani, Vivian Ruvolo, Hikari Taka, Naoko Kaga, Mika Kikkawa, Hajime Arai, Takashi Miida, Michael Andreeff, Paul A. Spagnuolo, Marina Konopleva

**Affiliations:** 1 Departments of Next Generation Hematology Laboratory, Juntendo University Graduate School of Medicine, Tokyo, Japan; 2 Section of Molecular Hematology and Therapy, Department of Leukemia, The University of Texas MD Anderson Cancer Center, Houston, TX USA; 3 Departments of Clinical Laboratory Medicine, Juntendo University Graduate School of Medicine, Tokyo, Japan; 4 Departments of Leading Center for the Development Research of Cancer Medicine, Juntendo University Graduate School of Medicine, Tokyo, Japan; 5 Division of Proteomics and BioMolecular Science, Juntendo University Graduate School of Medicine, Tokyo, Japan; 6 Departtment of Food Science, University of Guelph, Guelph, Ontario, Canada

## Abstract

Adipocytes are the prevalent stromal cell type in adult bone marrow (BM), and leukemia cells continuously adapt to deficiency of nutrients acquiring chemoresistant profiles in the BM microenvironment. We have previously shown that fatty acid metabolism is a key energy pathway for survival of acute myeloid leukemia (AML) cells in the adipocyte-abundant BM microenvironment. The novel fatty acid β-oxidation (FAO) inhibitor avocatin B, an odd-numbered carbon lipid derived from the avocado fruit, induced apoptosis and growth inhibition in mono-cultured AML cells. In AML cells co-cultured with BM adipocytes, FAO inhibition with avocatin B caused adaptive stimulation of free fatty acid (FFA) uptake through upregulation of *FABP4* mRNA, enhanced glucose uptake and switch to glycolysis. These changes reflect the compensatory response to a shortage of FFA supply to the mitochondria, and facilitate the protection of AML cells from avocatin B–induced apoptosis in the presence of BM adipocytes. However, the combination treatment of avocatin B and conventional anti-AML therapeutic agent cytarabine (AraC) increased reactive oxygen species and demonstrated highly synergistic effects on AML cells under BM adipocyte co-culture condition. These findings highlight the potential for combination regimens of AraC and FAO inhibitors that target bone marrow-resident chemoresistant AML cells.

## Introduction

The bone marrow (BM) microenvironment, which supports leukemia cell survival and chemotherapy resistance, presents an attractive target for novel therapeutic strategies. Recent research has identified numerous metabolic abnormalities in cancer, and metabolic modulation is evolving as a novel therapeutic approach^1–3^. Cancer cells are constantly adjusting their metabolic state in response to extracellular signaling and/or nutrient availability by making “decisions” such as quiescence, proliferation, or differentiation in a changing environment^3^. Leukemia cells encounter two major metabolic challenges: how to meet the bioenergetic and biosynthetic demands of increased cell proliferation and how to survive BM environmental fluctuations in external nutrient and oxygen availability. In fact, many tumor suppressors are known to support leukemic cell survival as metabolic regulators when essential metabolites become scarce^3^.

The incidence of acute myeloid leukemia (AML) increases with age, peaking in the 70 s^4^. The prognosis worsens with every decade of life starting at age 30–40 years, largely because older patients generally receive less intensive therapy due to comorbid conditions and the toxic side effects of existing chemotherapy^4^.There is an urgent need for novel therapeutic strategies in AML that are not only effective but can be tolerated by older patients.

Adipocytes are the prevalent type of stromal cells in adult, especially aging, BM, and fatty acids produced by adipocytes modulate the activity of signaling molecules^5^. Recent study demonstrated that the interplay between leukemic cells and adipose tissue created unique microenvironment supporting the metabolic demands and survival of a distinct leukemic stem cells (LSCs) subpopulation expressing the fatty acid transporter CD36^6^. Furthermore the *in vivo* finding of the higher rate of relapse after chemotherapy in obese leukemia mice than in normal-weight leukemia mice^7^ suggests the possibility that the increased adipocyte content of adult BM promotes leukemia growth and negatively affects sensitivity to chemotherapy. We previously reported that BM stromal cells promote AML cell survival via a metabolic shift from pyruvate oxidation to fatty acid β-oxidation (FAO), which causes mitochondrial uncoupling that diminishes mitochondrial formation of reactive oxygen species (ROS), decreases intracellular oxidative stress, and links to the Bcl-2 anti-apoptotic machinery^2,8^. Another study demonstrated that AML stem cells are unable to utilize glycolysis when mitochondrial respiration is inhibited, confirming that maintenance of mitochondrial function is essential for leukemia stem cell survival^9^. Furthermore, recent evidence suggests that the metabolic enzymes are often present in transcriptional complexes and play critical roles in determining transcriptional regulation providing a local supply of substrates/cofactors^10^.

**Figure 1:**
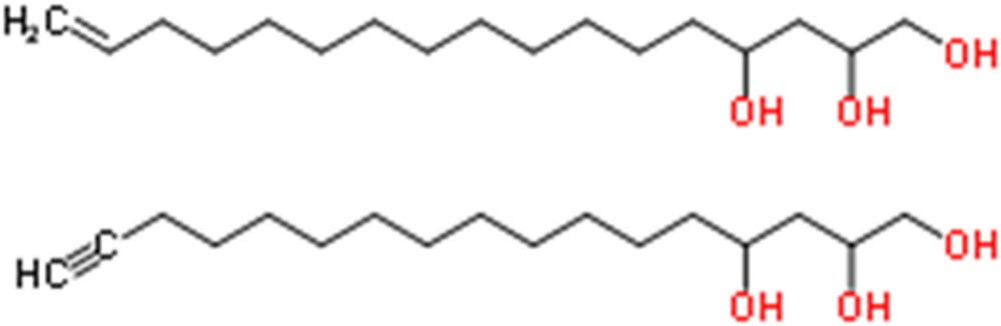
Structure of the fatty acid oxidation inhibitor avocatin B. Avocatin B is an odd-numbered carbon lipid with a 1:1 ratio of two 17-carbon lipids, derived from the avocado fruit.

In this study, we investigated the anti-leukemic efficacy and the molecular mechanisms of a novel small-molecule inhibitor of FAO, avocatin B, in AML cells. Avocatin B is an odd-numbered carbon lipid with a 1:1 ratio of two 17-carbon lipids that is derived from the avocado fruit and has been recently identified as a novel anti-AML compound (Fig. 1)^11^. We found that avocatin B upregulated the stress–induced transcription factor ATF4, AMPK signaling and reactive oxygen species (ROS). On the contrary, in AML cells co-cultured with BM adipocytes, an adaptive glucose uptake, glycolysis and free fatty acid (FFA) uptake was induced as the compensatory response to a shortage of FFA supply to the mitochondria, which reduced sensitivity of AML cells to avocatin B. We further demonstrated highly synergistic effects of avocatin B and cytarabine (AraC) causing ROS induction and apoptosis in AML cells under BM adipocyte co-culture conditions. These findings indicate that the BM adipocytes-induced AML protective effects can be abrogated by the combination of AraC and FAO inhibition, and the therapeutic potential of avocatin B in AML patients under adipocyte-enriched BM microenvironment.

## Results

### FAO inhibition by avocatin B increases fatty acid uptake by AML cells co-cultured with BM adipocytes

**Figure 2:**
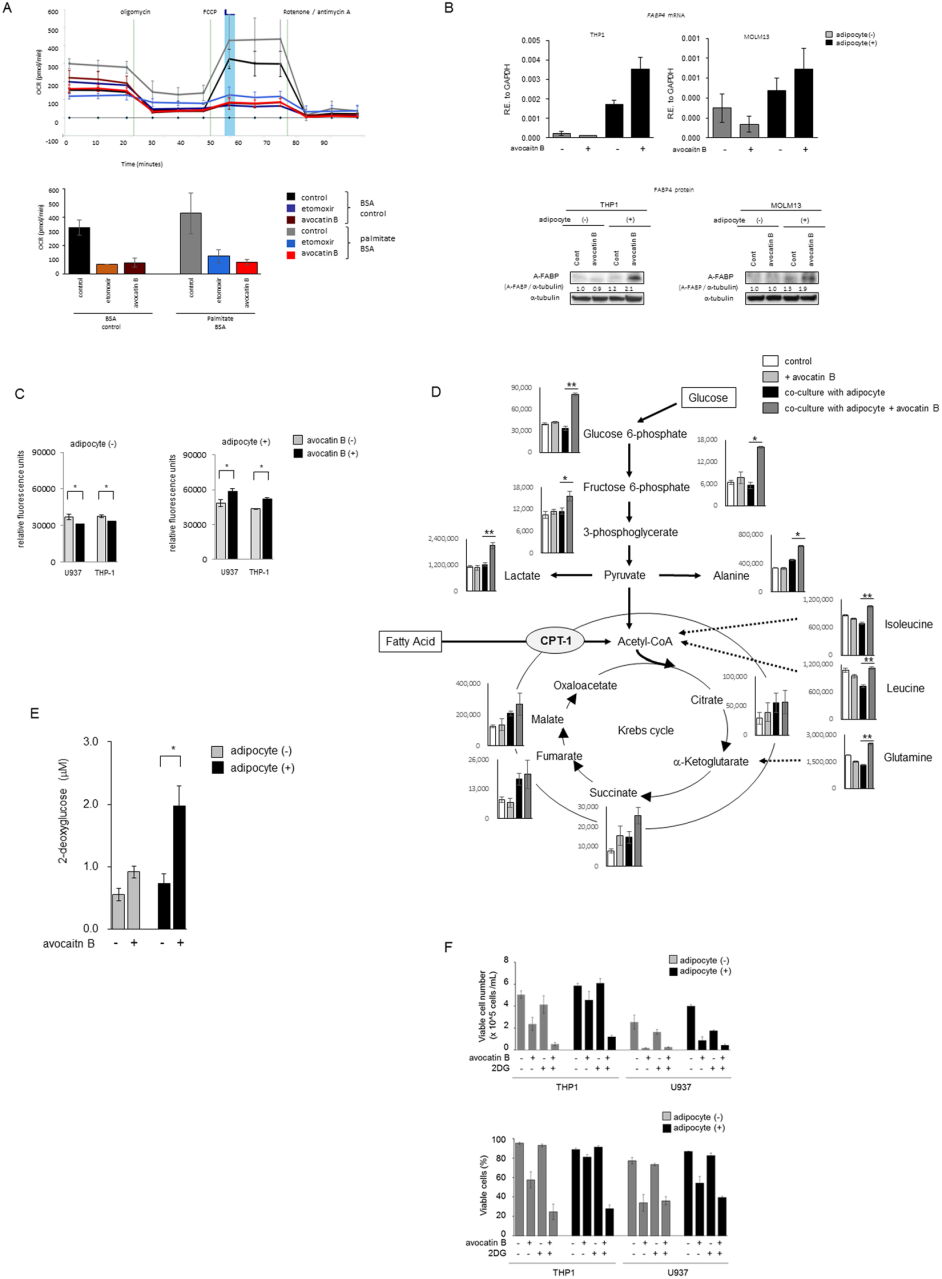
Avocatin B increases fatty acid uptake and glycolysis in THP-1 cells co-cultured with BM adipocytes. (**A**) Kinetic graph of the FAO/XF Cell Mito Stress Test assay of THP-1 cells, which determine the oxidization of exogeneous fatty acids; palmitate. The oxygen consumption rate (OCR) indicates the proportion of respiration that is supported by exogenous fatty acids under conditions of substrate limitation or with Palmitate-BSA substrate for exogenous fatty acids. Utilization of exogenous fatty acids was dependent on placing energetic stress via FCCP on the cells. Etomoxir (100uM) used as the positive control. Bar chart highlighting the differences in maximal respiration 50 minutes measurement (blue bar) which indicates utilization of exogenous fatty acids. Graphs show representative data from three independent experiments. (**B**) THP-1 and MOLM13 cells were cultured with or without avocatin B (10 μM) for 24 hours in the presence or absence of BM adipocytes. *FABP4* mRNA expression in the cells was determined by quantitative RT-PCR. The expression of transcripts of each gene relative (R.E.) to the expression of *GAPDH* transcripts was determined as described in Materials and Methods. Graphs show representative data from three independent experiments. A-FABP protein expression levels were detected by immunoblotting; Cont, controls. Results shown are representative of three independent experiments. (**C**) U937 and THP-1 cells were cultured with or without avocatin B (20 μM) for 2 hours in the presence or absence of BM adipocytes under serum-starved conditions and their fatty acid uptake assessed. Cells were plated at 50,000 cells/well, after which a fatty acid mixture (dodecanoic acid fluorescent fatty acid substrate) was added and the cells incubated for 1 hour. Fluorescent signal was measured with a plate reader using the bottom read mode. Graphs show the mean ± SD of the results from three independent experiments. *p < 0.05. (**D**) THP-1 cells were treated with avocatin B (10 μM) for 24 hours in the presence or absence of MSC-derived BM adipocytes under serum-starved conditions. Levels of metabolites in the cells were quantified by CE-TOF-MS analysis. The quantification data are superimposed on a metabolic pathway map that includes the glycolysis and Krebs pathways. Results shown are representative of three independent CE-TOF-MS experiments. Bars, SD. All *p*-values were determined by the Wilcoxon matched pair test. *p < 0.05; **p < 0.01. (E) THP-1 cells were cultured with or without avocatin B (20 μM) in the presence or absence of BM adipocytes under serum-starved conditions and glucose uptake measured. Plated cells were treated with avocatin B for 2 hours. Fluorescent signal was measured with a plate reader using the bottom read mode. Graphs show the mean ± SD of the results from three independent experiments. *p < 0.05. (F) THP-1 and U937 cells were treated with avocatin B (10 μM), 2DG (5 mM) or avocatin B + 2DG for 48 hours in the presence or absence of BM adipocytes under serum-starved conditions. The cell growth inhibition and cytotoxic effects were determined by cell counts using the trypan blue exclusion method. Graphs show the mean ± SD of the results from three independent experiments. *p < 0.05; **p < 0.01.

We initially examined induction of apoptosis by FAO inhibitor avocatin B in AML cells cultured alone or co-cultured with BM-derived adipocytes. Consistent with previously published data, avocatin B induced dose-dependent cell growth inhibition and cell death in AML cells^11,12^, however this anti-leukemia effects were suppressed by co-culture with BM-derived adipocytes (Supplementary Table S1). Inhibition of FAO by avocatin B was confirmed by the FAO/XF cell mito-stress test assay of THP-1 cells. Avocatin B induced marked repression of oxygen consumption rate (OCR) under conditions of substrate limitation and upon supplementation of the exogenous fatty acid Palmitate-BSA substrate (Fig. 2A). Avocatin B treatment significantly decreased the levels of FAO cycle enzymes HADHA, ACADVL, and ACADM^13^ in U937 cells co-cultured with BM adipocytes (Supplementary Fig. S1), demonstrating efficacious FAO inhibition under these conditions. We therefore focused on alternative mechanisms of AML cells survival after avocatin B treatment under BM adipocyte co-culture conditions. The pharmacologic inhibition of FAO by avocatin B is dependent on its entry into the mitochondria via CPT1, the ratelimiting enzyme regulating mitochondrial import of FFA^11^. Although *CPT1* mRNA expression was moderately increased after avocatin B treatment in the presence or absence of adipocytes, protein levels were only minimally affected by avocatin B (Supplementary Fig. S2, S3). Notably, avocatin B upregulated the expression levels of the lipid chaperone *FABP4* gene and protein^8,14^ under the FFA abundant BM adipocyte co-culture condition (Fig. 2B, Supplementary Fig. S3). Because FABP4 is a key factor implicated in adipocyte-tumor cell interaction and FAO metabolism^14,15^, we investigated whether avocatin B affected the uptake of FFA by AML cells. As shown in Fig. 2C, the FFA uptake was moderately but significantly suppressed by avocatin B treatment in mono-cultured U937 and THP-1 cells. However, avocatin B increased FFA uptake significantly in both cell types when co-cultured with BM adipocytes. These results indicate that FAO inhibition by avocatin B reciprocally stimulates feedback FFA uptake in AML cells exposed to BM adipocytes.

### Avocatin B increases glycolysis in AML cells co-cultured with BM adipocytes

We next investigated the metabolic changes induced by avocatin B in AML cells in the presence or absence of BM adipocytes. CE-TOF-MS analyses identified 98 and 87 metabolites in two independent experiments of THP-1 cells (Supplementary Table S2). As shown in Fig. 2D, avocatin B increased glucose 6-phosphate and fructose 6-phosphate, the dominant products of imported glucose, in THP-1 cells co-cultured with BM adipocytes but not in the mono-cultured cells. The increased glucose uptake by avocatin B in THP-1 cells co-cultured with BM adipocytes was confirmed by glucose cellular uptake measurement (Fig. 2E). Similar findings were observed in U937 cells co-cultured with BM adipocytes (data not shown). Increased glucose uptake by avocatin B was associated with higher levels of lactate production and increase in alanine (Fig. 2E), consistent with activation of glycolysis and glucose utilization. These results indicate that compensatory glycolysis in the setting of FAO inhibition by avocatin B could contribute towards survival of AML cells exposed to BM adipocytes. Indeed, a glycolysis inhibitor 2-Deoxy-D-glucose (2DG) enhanced the cell growth inhibition and cytotoxic effects of avocatin B under both monoculture and adipocytes co-culture conditions in THP-1 and U937 cells (Fig. 2F). Of notes, these combinational effects were more prominent in BM adipocyte co-culture conditions. Intra-cellular levels of Krebs cycle intermediates succinate, fumarate and malate were also upregulated by co-culture with BM adipocytes. Treatment with avocatin B was associated with increased levels of amino acids in AML cells, possibly due to increased catabolism which can in turn facilitate maintenance of Krebs cycle (Fig. 2C, Supplementary Table S2).

### Avocatin B induces ATF4 activation in AML cells co-cultured with BM adipocytes

**Figure 3:**
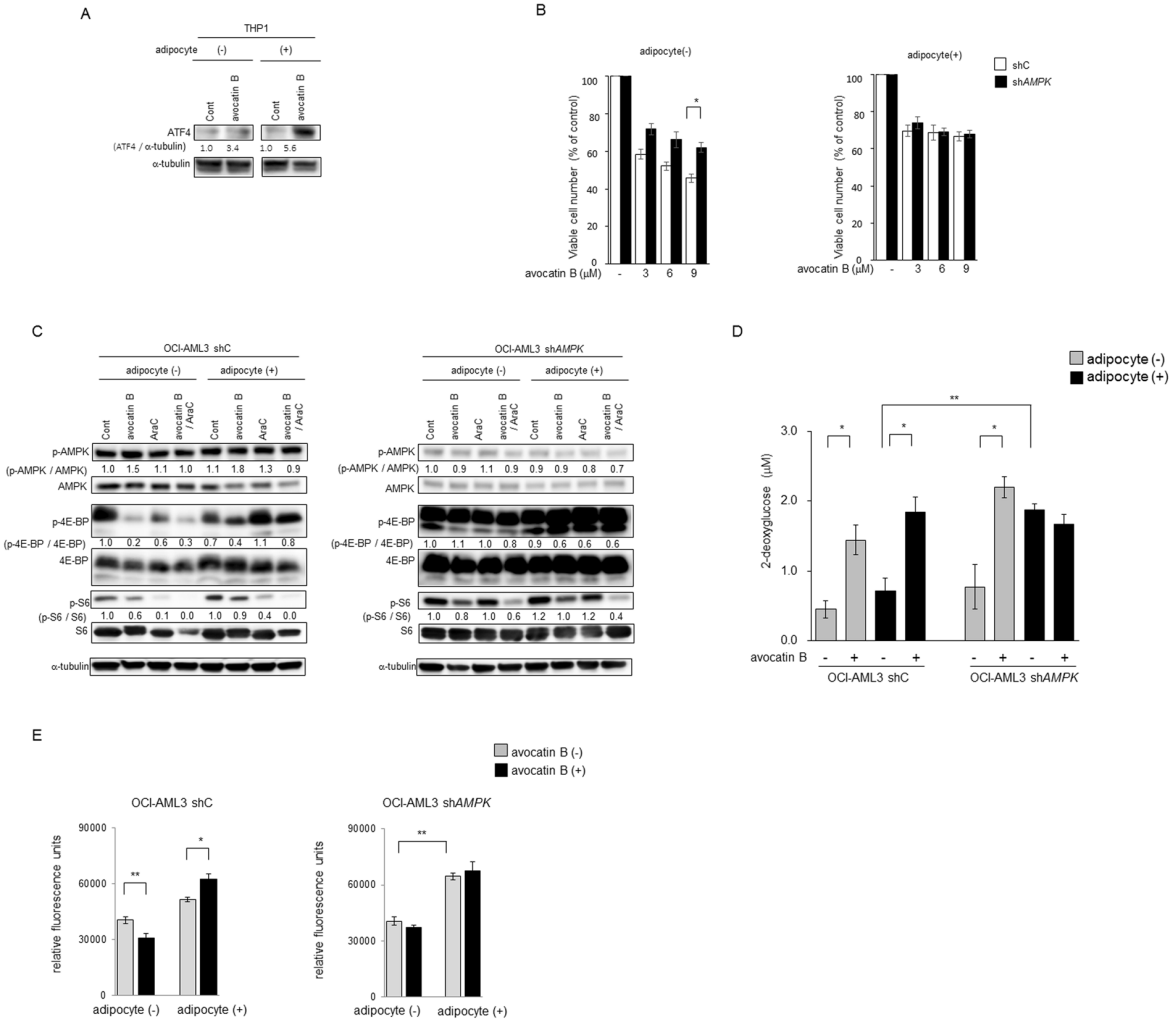
Avocatin B increases ATF4 activation and AMPK-mTOR signaling in AMfL cells co-cultured with BM adipocytes. (**A**) THP-1 cells were co-cultured with BM adipocytes for 24 hours with or without avocatin B (10 μM), and expression levels of ATF4 protein were detected by immunoblotting; Cont, controls. Results shown are representative of three independent experiments. (**B**) OCI-AML3 cells transfected with control short hairpin RNA (shC) or shRNA against *AMPK* (sh*AMPK*) were treated with the indicated concentrations of avocatin B for 48 hours in the presence or absence of BM adipocytes under serum-starved conditions. The effects on cell viability were determined by cell counts using the trypan blue exclusion method. Graphs show the mean ± SD of the results from three independent experiments. (**C**) OCI-AML3 cells transfected with control short hairpin RNA (shC) or shRNA against *AMPK* (sh*AMPK*) were cultured with or without avocatin B (10 μM) and AraC (3 μM) for 18 hours in the presence or absence of BM adipocytes. Expression levels of AMPK, p-AMPK, 4E-BP1, p-4E-BP1, S6, p-S6 and α-tubulin proteins in the cells were detected by immunoblotting; Cont, controls. Results shown are representative of three independent experiments. (**D**) OCI-AML3 cells transfected with control short hairpin RNA (shC) or shRNA against *AMPK* (sh*AMPK*) were cultured with or without avocatin B (20 μM) in the presence or absence of BM adipocytes under serum-starved conditions and glucose uptake measured. Plated cells were treated with avocatin B for 2 hours. Fluorescent signal was measured with a plate reader using the bottom read mode. Graphs show the mean ± SD of the results from three independent experiments. *p < 0.05. (**E**) OCI-AML3 cells transfected with control short hairpin RNA (shC) or shRNA against *AMPK* (sh*AMPK*) were cultured with or without avocatin B (20 μM) for 2 hours in the presence or absence of BM adipocytes under serum-starved conditions. Cells were plated at 50,000 cells/well, after which fatty acid (FA) uptake was determined by adding a fatty acid mixture (dodecanoic acid fluorescent fatty acid substrate) and incubating for 1 hour. Fluorescent signal was measured with a plate reader using the bottom read mode. Graphs show the mean ± SD of the results from three independent experiments. **p < 0.01; *p < 0.05.

To investigate the transcriptional changes induced by avocatin B in AML cells co-cultured with BM adipocytes, we profiled gene expression by DNA microarray analysis using THP-1 cells; treated with avocatin B (10 μM) for 24 hours in the presence of MSC-derived BM adipocytes under serum-starved conditions. This analysis detected the consistent upregulation of 45 genes and downregulation of 58 genes (>2.0 fold in both cases) in THP-1 cells by co-culture with BM adipocytes in two independent experiments. As shown in Table 1, the Ingenuity Pathway Analysis bioinformatics tool^16^ highlighted activation of the potent upstream regulators CXCL12, STAT3, MAPK, and NFκB. In turn, avocatin B treatment upregulated 71 genes and downregulated 27 genes in THP-1 cells co-cultured with BM adipocytes in two independent experiments. The stress response genes *ASNS, DDIT3, DDIT4, SESN2, PCK2, PHGDH, PSAT1* and *STC2*, which are the downstream targets of transcription factor ATF4, the master regulator of the endoplasmic reticulum (ER) stress response, have been upregulated by avocatin B treatment under co-culture conditions (Table 1). Immunoblot analysis confirmed that avocatin B markedly upregulated ATF4 expression in THP-1 cells in the presence but not in the absence of BM adipocytes (Fig. 3A, Supplementary Fig. S4). These findings indicate that FAO inhibition by avocatin B induced ER stress and stimulated ATF4 in AML cells co-cultured with BM adipocytes.

**Table 1:** Upstream regulators detected in THP-1 cells in mono-culture or co-cultured with BM adipocytes with or without avocatin B treatment.

Upstream Regulator	microarray 1		microarray 2		Target molecules in dataset
	Activation z-score	p-value	Activation z-score	p-value	
**BM-adipocytes co-culture**					
Activated					
TNF	5.100	<0.001	3.333	<0.001	ALOX5AP, BCL2A1, BCL3, BCL6, BHLHE40, BTG2, C3, CCL2, CCL20, CCL3L3, CCR1, CD163, EMP1, FCGR2B, FN1, FPR1, HIPK2, ICAM1, IER3, IFI16, IFITM1, IL1B, IL4R, ITGB3, KLF10, MMP14, MMP9, MUC1, OLR1, PIM1, PLAUR, PLOD2, PPARD, RGS1, RGS16, SERPINB10, SGK1, SLC11A1, SLC20A1, SOCS3, SPP1, TGFBR1, TGM2, TMEM176B, TNF, TREM1, TREM2, VASH1, ZFP36
CSF2	4.533	<0.001	3.397	<0.001	ALOX5AP, BCL2A1, BCL3, C3, CCL2, CCL3L3, CCR1, CD180, CLEC7A, FAM65B, FCGR2B, ICAM1, IER3, IL1B, ITGB3, MMP14, MMP9, PIM1, SGK1, SOCS3, SPP1, TARP, TGM2, TNF, TREM1, ZFP36
PDGF BB	4.500	<0.001	2.900	<0.001	BCL3, BHLHE40, C3, CCL2, CCL20, FN1, IER3, IL1B, KLF10, MMP14, MMP9, OLR1, PIM1, PPARD, RGS1, SGK1, SLC2A3, SOCS3, TGM2, TRIB1, VCAN, ZFP36
IL1B	4.495	<0.001	3.785	<0.001	A2M, BCL2A1, BCL3, BTG2, C3, CCL2, CCL20, CCR1, FAM129A, FCGR2B, FN1, ICAM1, IER3, IFI16, IL1B, ITGB3, KLF10, MMP14, MMP9, MUC1, OLR1, PIM1, PLXDC2, RGS16, S100A10, SLC20A1, SOCS3, SPP1, TGM2, TMEM176B, TNF, TREM1, TREM2, VCAN, ZFP36
IL6	4.426	<0.001	3.376	<0.001	A2M, ADGRE1, BCL3, BCL6, BTG2, C3, CCL2, CCL20, CCL3L3, CCR1, CD163, FN1, ICAM1, IFI16, IL4R, ITGB3, MMP9, MUC1, PIM1, SGK1, SOCS3, SPP1, TGM2, TLR6, TNF
TNFSF11	3.940	<0.001	3.173	<0.001	ADGRE1, BCL2A1, CCL2, CCL3L3, CCR1, FPR1, HIPK2, ICAM1, IER3, IL1B, ITGB3, MMP9, OCSTAMP, PIM1, PLAUR, RGS16, SLC20A1, SOCS3, SPP1, TNF
ERK	3.912	<0.001	2.594	<0.001	BTG2, CCL2, CCR1, FN1, ICAM1, IER3, IL1B, ITGB3, KLF10, MMP14, MMP9, MUC1, PPARD, TGM2, TNF, VCAN, ZFP36
AGT	3.900	<0.001	2.309	<0.001	ADGRE1, ANXA3, CCL2, CCL3L3, FN1, ICAM1, IL1B, ITGB3, MMP9, OLR1, SGK1, SOCS3, SPP1, TGFBR1, TNF, ZFP36
TGFB1	3.874	<0.001	3.185	<0.001	ALOX5AP, BCL3, BCL6, BHLHE40, CCL2, CCL20, CCL3L3, CCR1, CD163, DOCK4, FN1, ICAM1, IER3, IFI16, IL1B, IL4R, ITGB3, KCNQ3, KLF10, MMP14, MMP9, OLR1, PIM1, PLAUR, PLOD2, PPARD, RGCC, S100A10, SERPINB10, SGK1, SKIL, SLC20A1, SLC2A3, SOCS3, SPP1, SPRY1, TGFBR1, TGM2, TNF, VCAN, ZFP36
NFkB (complex)	3.781	<0.001	2.772	<0.001	A2M, ADGRE1, BCL2A1, BCL3, C3, CCL2, CCL20, CCL3L3, FN1, ICAM1, IER3, IL1B, MMP9, OLR1, RGS16, SOCS3, SPP1, TGM2, TNF, ZFP36
P38 MAPK	3.597	<0.001	2.741	<0.001	BCL2A1, CCL2, CCL3L3, FN1, ICAM1, IER3, IL1B, ITGB3, MMP9, PLAUR, SGK1, SOCS3, TNF, TREM1, TRIB1, ZFP36
STAT3	3.537	<0.001	3.391	<0.001	A2M, BCL3, BCL6, CCL2, CCL20, CCL3L3, CCR1, FN1, ICAM1, IFI16, IFITM1, IL1B, IL4R, MMP9, MUC1, PIM1, PLAUR, SGK1, SOCS3, TNF, VCAN, ZFP36
EGF	3.502	<0.001	2.286	<0.001	BTG2, CCL20, FN1, ICAM1, IER3, IL1B, ITGB3, KLF10, MMP9, MUC1, PLAUR, PPARD, S100A10, SOCS3, SPP1, TGM2, VCAN, ZFP36
TLR3	3.347	<0.001	2.393	<0.001	C3, CCL2, CCL20, CCL3L3, IER3, IFI16, IL1B, RGS1, SOCS3, SPRY1, TNF, TREM1
IL17A	3.277	<0.001	2.393	<0.001	BCL2A1, C3, CCL2, CCL20, CD163, ICAM1, IL1B, MMP9, RGS16, SOCS3, TNF
TLR4	3.131	<0.001	2.403	<0.001	C3, CCL2, CCL3L3, CD163, FCGR2B, FGL2, ICAM1, IFI16, IL1B, MMP9, RGCC, RGS1, RGS16, SOCS3, SPP1, TNF, TREM1, TREM2
STAT4	3.118	<0.001	2.204	<0.001	BCL3, C3, CCL2, IER3, PLOD2, RGCC, RGS16, SLC2A3, SOCS3, TNF
Inhibited					
COL18A1	−2.200	<0.001	−2.213	1.96E-05	CCL2, FN1, ICAM1, ITGB3, TNF, FOS, JUN, JUNB, MCL1, NFKBIA, PIM1, SOCS3
SOCS1	−2.946	<0.001	−2.000	5.53E-04	A2M, BCL2A1, CCL2, ICAM1, IFI16, IL1B, IL4R, DUSP1, FOS, JUN, MUC1, SOCS3, TNF
**Avocatin B treated under BM-adipocytes co-culture**					
Activated					
ATF4	2.772	<0.001	2.171	<0.001	ASNS, CHAC1, DDIT3, DDIT4, PCK2, PHGDH, PSAT1, SLC6A9, SLC7A5, STC2
inhiited					
TRIB3	−2.401	<0.001	−2.186	<0.001	ASNS, DDIT3, DDIT4, PCK2, PSAT1, STC2

### AMPK and-mTOR modulate sensitivity to avocatin B

In a previous study, we demonstrated that AMPK activation supports AML cell survival during nutrient starvation in the presence of BM adipocytes^8^. Because AMPK is known to promote metabolic homeostasis by modulating fatty acid uptake and oxidation^17^, glucose uptake^18,19^ and glycolysis^20,21^, we utilized *AMPK* knockdown AML cells^8^ to investigate the role of AMPK in response to avocatin B^22^. In OCI-AML3 cells with stable knockdown of *AMPK* (sh*AMPK*) expressed increased phosphorylaton of cap-dependent translation repressor 4E-BP1 (p-4E-BP1) and ribosomal protein S6 (p-S6), downstream targets of mTOR (Fig. 3B). In parental shC OCI-AML3 cells, avocatin B treatment mildly increased AMPK activation and reduced p-4E-BP1 and p-S6 expressions in mono-cultured cells, which was partially reversed by co-culture with BM adipocytes along with decreased sensitivity against avocatin B (Fig. 3B, C, Supplementary Fig. S5). sh*AMPK* OCI-AML3 cells were less sensitive against avocatin B compared to shC OCI-AML3 cells in mono-culture condition (Fig. 3C, Supplementary Fig. S5). Adipocyte co-culture reversed growth-inhibitory effects of avocatin B irrespective of AMPK levels (Fig. 3C, Supplementary Fig. S5). In sh*AMPK* OCI-AML3 cells avocatin B treatment minimally reduced p-S6 overexpression irrespective of culture conditions, and overexpressed p-4E-BP1 was even further increased by avocatin B in the presence of BM adipocytes (Fig. 3C). These findings indicate that avocatin B induced AMPK activation, and suppression of its downstream mTOR signaling could at least partially contribute to AML growth inhibition.

As shown in Fig. 3D, under serum starved condition, *AMPK* knockdown OCI-AML3 cells with high levels of p-4E-BP1 and p-S6 showed higher baseline glucose uptake than parental OCI-AML3 cells, which was further increased by avocatin B treatment in monoculture condition. Of note, under BM adipocyte co-culture condition *AMPK* knockdown OCI-AML cells increased glucose uptake that was not affected by avocatin B treatment. Regarding FFA uptake, avocatin B decreased FFA uptake in parental OCI-AML3 cells under mono-culture condition but increased in co-culture with BM adipocytes (Fig. 3E), concordant with the finding in U937 cells (Fig. 2C). In *AMPK* knockdown OCI-AML3 cells avocatin B did not stimulate FFA uptake regardless in the absence or presence of BM adipocytes (Fig. 3E). These results indicate that AMPK at least partially modulates FFA uptake in response to avocatin B–induced FAO suppression.

### Avocatin B combined with AraC synergistically induces cell death in AML cells co-cultured with BM adipocytes

**Figure 4:**
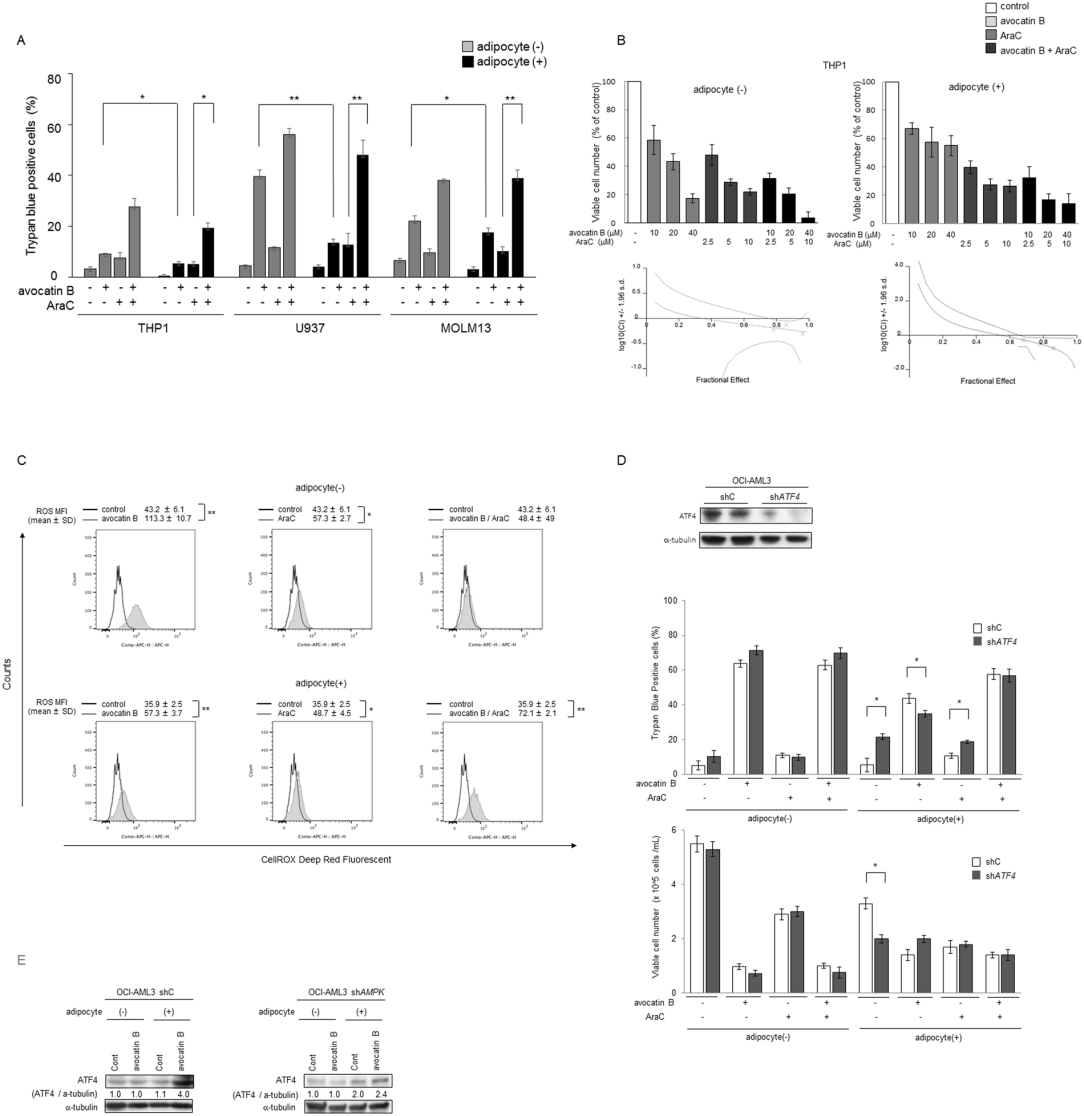
Effects of avocatin B and AraC on AML cells in presence and absence of co-cultured BM adipocytes. (**A**) THP-1, U937, and MOLM13 cells were treated with avocatin B (10 μM for THP-1 and U937, 7 μM for MOLM13), AraC (3 μM for THP-1, 0.1 μM for U937, 3 μM for MOLM13) or avocatin B + AraC for 48 hours in the presence or absence of MSC-derived BM adipocytes. Cells were cultured in medium containing 5% FBS. Percentages of cell death were determined by cell counts using the trypan blue exclusion method. Graphs show the mean ± SD of the results from three independent experiments. *p < 0.05; **p < 0.01. (**B**) THP-1 cells were treated with the indicated concentrations of avocatin B, AraC, or avocatin B + AraC for 48 hours in the presence or absence of BM adipocytes. Percentages of viable cells compared to the control condition were determined by cell counts using the trypan blue exclusion method. Graphs show the mean ± SD of the results from three independent experiments. Combination index (CI) values which assess drug-interaction effects, were then calculated using the calcusyn software^40^, as described in the text. CI values of < 1, > 1 or equal to 1 denote statistical synergy, antagonism, or additivity, respectively. Representative figures shown. (**C**) U937 cells were treated with/without avocatin B (10 μM), AraC (0.1 μM) or avocatin B + AraC for 24 hours in the presence or absence of BM adipocytes under serum-starved conditions. Representative histograms of CellROX staining (ROS production) in the viable cells (SYTOX staining) under the indicated conditions are shown. Mean fluorescence intensity (MFI) indicates the mean ± SD of results of three independent experiments. *p < 0.05, **p < 0.01. (**D**) OCI-AML3 cells transfected with control short hairpin RNA (shC) or shRNA against *ATF4* (sh*ATF4*) were cultured for 48 hours with or without avocatin B (9 μM) and AraC (3 μM) in the presence or absence of BM adipocytes under serum-starved conditions, then the cytotoxic effects and cell growth inhibition were determined by cell counts using the trypan blue exclusion method. Graphs show the mean ± SD of the results from three independent experiments. *p < 0.05. (**E**) OCI-AML3 cells transfected with control short hairpin RNA (shC) or shRNA against *ATF4* (sh*ATF4*) were co-cultured with BM adipocytes for 24 hours with or without avocatin B (10 μM), and expression levels of ATF4 protein were detected by immunoblotting; Cont, controls. Results shown are representative of three independent experiments.

Here, we observed that the apoptotic efficacy of AraC was significantly enhanced by avocatin B in AML cells under both, medium only and under BM adipocyte co-culture conditions (Fig. 4A). The synergy of avocatin B and AraC-induced growth inhibition was formally demonstrated in THP-1 cells using Chou-Talalay method, with combination index of 0.7 in mono-culture and of 0.4 in adipocyte co-culture conditions (Fig. 4B, Supplementary Table S3). Because FAO inhibition can cause ROS generation leading to progressive redox damage, we examined ROS levels of avocatin B treated U937 cells cultured in the presence or absence of BM adipocytes. Co-culture with BM adipocytes decreased constitutive ROS production (Supplementary Fig. S6). In turn, treatment with avocatin B increased ROS in U937 cells, indicating that avocatin B causes oxidative damage in AML cells (Fig. 4C). Although avocatin B induced ROS was more prominent in the mono-cultured U937 cells than in the BM adipocyte co-cultured cells, combined treatment with AraC enhanced ROS only in the BM adipocyte co-cultured condition (Fig. 4C, Supplementary Fig. S7).

Because we observed avocatin B-induced upregulation of ATF4 in AML cells in the presence but not in the absence of BM adipocytes, we performed *ATF4* knockdown experiments and examined the cytotoxic efficacy of avocatin B with/without AraC combination in the presence of BM adipocytes (Fig. 4D, Supplementary Fig. S8). In mono-culture condition stable knockdown of *ATF4* (sh*ATF4*) and control shC OCI-AML3 cells showed no significant difference in spontaneous and avocatin B or AraC–induced cell death and cell growth inhibition. On the other hand, in BM adipocyte co-culture condition control OCI-AML3 cells demonstrated higher sensitivity to avocatin B, but lower sensitivity to AraC-induced cell death compared to sh*ATF4* cells, which was reversed by the combination with avocatin B (Fig. 4D, Supplementary Fig. S8). These findings indicate that ATF4 activation facilitates apoptosis induction by avocatin B in AML cells in the presence of BM adipocytes.

With respect to the role of AMPK, AraC/avocatin B co-treatment further inhibited S6 phosphorylation in OCI-AML3 cells, which was abrogated by *AMPK* knockdown (Fig. 3C). Intriguingly, avocatin B induced ATF4 expression in parental OCI-AML3 cells was abrogated in sh*AMPK* OCI-AML3 cells under co-culture condition with BM adipocytes (Fig. 4E, Supplementary Fig. S9).

## Discussion

In this study, we studied the activity and metabolic consequences of FAO inhibition with avocatin B, alone and in combination with AraC, under adipocytes abundant conditions of BM microenvironment where adipose-resident LSCs exhibit a pro-inflammatory phenotype and induce lipolysis in BM adipocytes, which fuels fatty acid oxidation in leukemic cells^6^. Our findings indicate that while FAO inhibition alone triggers metabolic adaptation and reduced cytotoxicity, it favors highly synergistic interactions with conventional anti-AML therapeutic agent AraC.

Because AML cells have been shown to contain higher mitochondrial mass than normal cells^23^ and depend on fatty acid substrates for survival^2^, blocking FAO was proposed as one of the potential therapeutic strategies. Several studies demonstrated that CPT1^2,24^ and a plasma membrane carnitine transporter CT2^25^ are putative targets for FAO inhibition. Avocatin B, an odd-numbered carbon lipid, is not efficiently oxidized and is more slowly catabolized than even-numbered fatty acids^26–28^. Although we previously reported that avocatin B induced dose-dependent cell growth inhibition and cell death in AML cells^11,12^, this study demonstrated that the anti-leukemia effects of avocatin B was suppressed by co-culture with BM-derived adipocytes. However, avocatin B showed synergistic apoptotic effect when AML cells were co-treated with AraC, under conditions of adipocyte co-cultures with ER stress-induced ATF4 activation and increased ROS production. To elucidate the alternative mechanisms of pro- and anti-apoptotic role of FAO inhibition by avocatin B, we focused on the compensatory response to metabolic stress. The energetic stress of FAO inhibition by avocatin B induced upregulation of FFA, and additionally glucose uptake and glycolysis in AML cells co-cultured with BM adipocytes, which might counteract apoptosis.

Avocatin B treatment increased FFA uptake and upregulated FA chaperone *FABP4* mRNA and protein expressions in AML cells co-cultured with BM adipocytes. Concordant with our observation, it has been reported that the *Abcb11*-knockout (KO) mice deficient in mitochondrial FAO displayed increased expression of FABP4 along with a FA importer CD36, enhanced free FA uptake and mitochondrial import without FAO in liver^29^. These findings could reflect a feedback system induced by shortage of FFA supply for FAO to the mitochondria.

In addition, avocatin B increased glucose uptake and stimulated glycolysis in cells co-cultured with BM adipocytes. These compensatory metabolic pathways may provide continued supply of ATP to AML cells under energetic stress of FAO inhibition, reducing anti-AML activity of avocatin B. Consistent with this notion, the combinatorial inhibition of glycolysis by 2DG enhanced avocatin B-induced cell growth inhibition and cytotoxicity in AML cells, more prominent under BM adipocyte co-culture than in monoculture conditions. It is possible, however, that the hydrophobic nature of avocatin B might cause its accumulation in the adipocytes and impact the results of the co-culture model, to be studied in the future.

In AML cells co-cultured with BM adipocytes, avocatin B significantly increased expression of transcription factor ATF4, the master regulator of the integrated stress response, and its downstream target genes, including *ASNS*. Ye *et al*. demonstrated that activation of the ATF4-ASNS pathway supports tumor cell survival under stress conditions, including nutrient deprivation^30^. Paradoxically, however, ATF4 is also known to positively regulate expression of genes involved in apoptosis^31–33^. Notably, *ATF4* knockdown caused more spontaneous or AraC induced apoptosis but decreased apoptosis induction by avocatin B treatment in AML cells under BM adipocyte co-culture conditions. These results indicate that activation of ATF4 contributed to pro-apoptotic effects of avocatin B, concordant with the role of ATF4 as a positive regulator of apoptosis^30^. Under BM adipocyte co-culture condition, ATF4 activation by avocatin B likely contributes towards apoptosis induction by avocatin B/AraC combination treatment even though the apoptotic effects of a single agent AraC are repressed by ATF4 itself.

**Figure 5:**
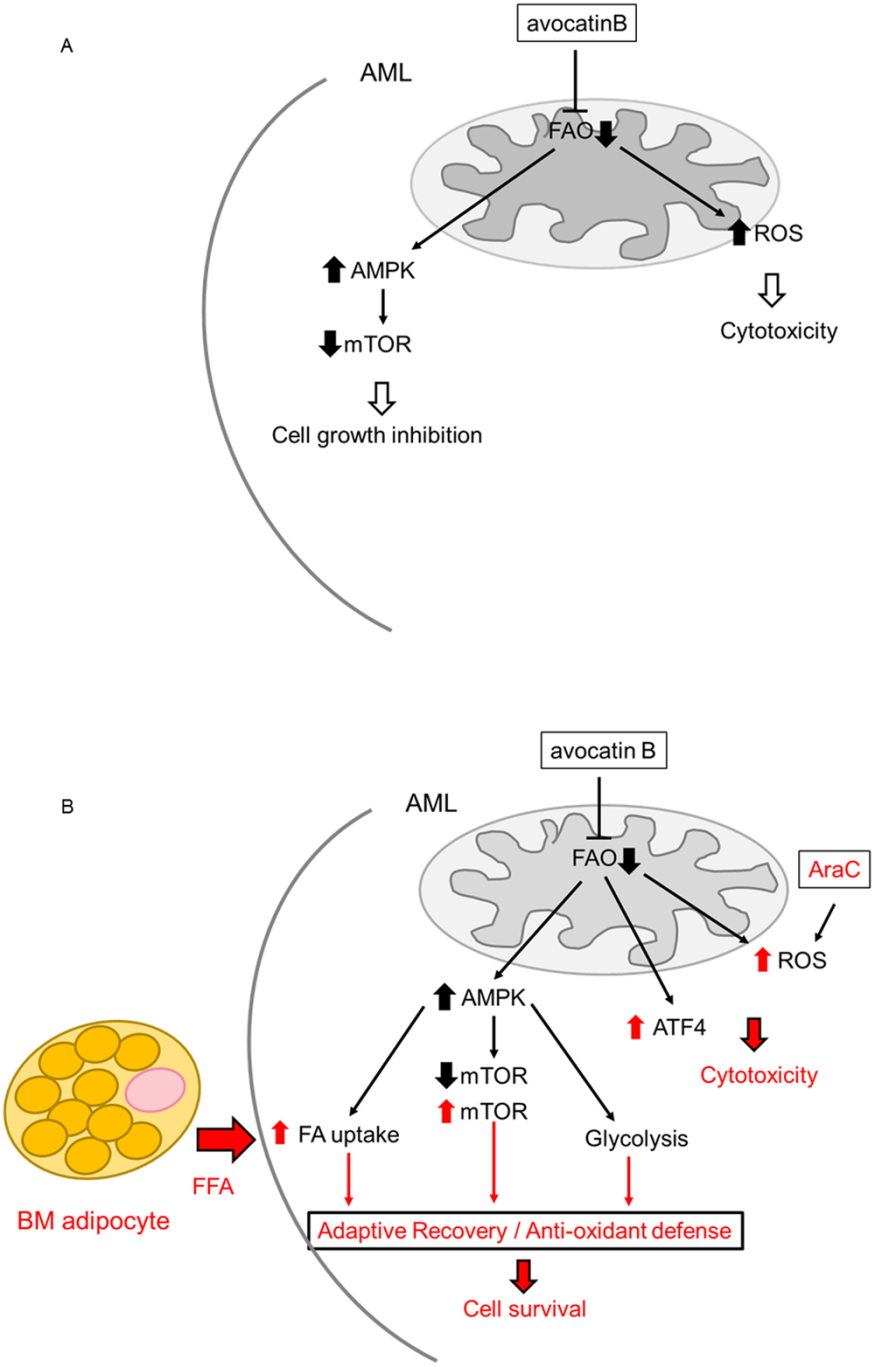
Schematic diagram illustrating the mediators involved in the adaptation to ROS stress and cell death induced by avocatin B and AraC in AML cells co-cultured with BM adipocytes. (**A**) Energetic stress triggered by the inhibition of FAO with avocatin B increases ROS and promotes activation of AMPK pathway that represses mTOR, which results in cytotoxicity and cell growth inhibition. (**B**) In the adipocyte-abundant BM microenvironment, the adaptive mechanisms upon FAO inhibition by avocatin B, including increase of FA (fatty adic) uptake, upregulation of glycolysis, and positive modification of mTOR signaling might contribute to survival of AML. In turn, AraC combination with avocatin B might associate with heightened ROS induction. Avocatin B also upregulates stress induced ATF4 in AML cells co-cultured with BM adipocytes. FAO inhibition which suppressed oxidative phosphorylation resulted in sensitization to AraC. These findings and our results indicate that the increased dependence on FAO metabolism of the AraC exposed AML cells might be responsible for a synergistic apoptotic effect of avocatin B with AraC under conditions of adipocyte co-cultures. Indeed, we observed that avocatin B and AraC combination treatment increased ROS production only in adipocyte co-cultured AML cells but not in mono-cultured cells. FFA; free fatty acid.

The regulatory interaction between mTORC1 and ER stress is known as bidirectional crosstalk^34^. AMPK inhibits the mTOR pathway and supports tumor cells escape from the stress-induced cell death in unfavorable environments such as deficient in oxygen and nutrients^35^. In our system, OCI-AML3 cells with *AMPK* knockdown and mTOR signaling hyperactivation were less sensitive to avocatin B than parental cells under BM adipocyte co-culture conditions. These results suggest that AMPK-mTOR signaling are involved in the mechanisms whereby AML cells are protected, at least in part, from avocatin B–induced apoptosis in the presence of BM adipocytes (Fig. 5).

Very recently, Farge *et al*.^1^ demonstrated that AraC resistant AML cells exhibited increased FAO and a high mitochondrial oxidative phosphorylation status, and reported that FAO inhibition which suppressed oxidative phosphorylation resulted in sensitization to AraC. These findings and our results indicate that the increased dependence on FAO metabolism of the AraC exposed AML cells might be responsible for a synergistic apoptotic effect of avocatin B with AraC under conditions of adipocyte co-cultures. Indeed, we observed that avocatin B and AraC combination increased ROS production only in adipocyte co-cultured AML cells but not in mono-cultured cells. However, the functional importance of the increased ROS in the execution of cell death remains to be elucidated. The primary cells experiments also warrant further investigation.

In a previous study, we demonstrated that in AML cells co-culture with BM adipocytes induced downregulation of phospho (p-) Akt in contrast to findings of p-Akt upregulation in the cells co-cultured with MSCs^36^. These findings suggest that distinct mechanism(s) are operational in BM adipocyte and MSCs co-culture systems, which are both involved in apoptosis resistance in leukemia cells.

In conclusion, the findings of this study indicate that various adaptive mechanisms upon FAO inhibition, including upregulation of glycolysis and AMPK-mTOR signaling might contribute to survival of AML in the adipocyte-abundant BM microenvironment. These results shed the light on limited efficacy of “metabolic” inhibitors when used as single agents, due to reciprocal activation of the bypass metabolic pathways. In turn, FAO inhibition combined with AraC is associated with heightened ROS induction and synergistic apoptosis under conditions mimicking aged bone marrow microenvironment. This study highlights the importance of studying AML cells in the relevant microenvironmental context and emphasizes the potential of drug combination regimens targeting residual chemoresistant AML cells in the BM.

## Materials and Methods

### Cell lines, primary samples, and culture conditions

The U937 and THP-1 cells (ATCC, Manassas, VA) and MOLM13 and OCI-AML3 cells (DSMZ, Braunschweig, Germany) were cultured in RPMI 1640 medium with 10% FBS. Mesenchymal stem cells (MSCs) were obtained from BM from healthy donors (aged 20 to 54 years), who gave informed consent in accordance with institutional guidelines set forth by The University of Texas MD Anderson Cancer Center per Declaration of Helsinki principles. The experimental protocol was approved by the Institutional Review Board of University of Texas MD Anderson Cancer Center (Protocol PA13-1025).

MSCs were cultured in minimum essential medium alpha supplemented with 20% FBS. Passage 2 MSCs that comprised a single phenotypic population as previously described^37^ and reached 90% confluence were allowed to differentiate to adipocytes, which were identified by morphology and by the presence of lipid droplets that stained with oil red O^37^. For this study, we utilized the monolayer culture composed of more than 50% BM adipocytes. For co-culture experiments, leukemia cells were co-cultured by plating them on top of BM adipocytes in serum-free conditions and later separated from the BM adipocyte monolayer by careful pipetting with ice-cold PBS, repeated twice. The purity of the leukemic cells separated from adipocytes was confirmed by the absence of *CD90* mRNA expression by PCR. Cell were treated with FAO inhibitor avocatin B^11^ (Fig. 1), glycolysis inhibitor 2-Deoxy-D-glucose (2DG, Wako Pure Chemical Industries, Osaka, Japan) and/or AraC (Nippon Shinyaku, Kyoko, Japan). Short hairpin RNA (shRNA) targeting isoform α1 of the AMPK catalytic subunit (targeting residues 1621–1641 of RefSeq NM_006251.5) and shRNA targeting ATF4 (targeting residues 1495 to 1515 of RefSeq NM_001675.3) were obtained from GE Healthcare Biosciences (Pittsburgh, PA).

### Cell viability and proliferation

Cell viability and proliferation were assessed by the trypan blue exclusion method using a Vi-Cell XR cell counter (Beckman-Coulter, Brea, CA).

### Extracellular flux assays

FAO inhibition of avocatin B was assessed by an extracellular flux analyzer using the XF Palmitate-BSA FAO Substrate (Seahorse Bioscience, North Billerica, MA) accordance with the manufacturer’s instructions. Individual wells of an XF24 cell culture microplate were coated with CellTak and THP-1 cell (5 × 105) were plated in RPMI-1640 and 10% human serum in six-well plates with or without avocatin B or etomoxir (positive control) for 2 h. Cells were counted and plated onto XF microplates. RPMI-1640 medium was replaced with XF base and measure cellular oxygen consumption rates (OCR) media as recommended by Seahorse Bioscience. Cellular oxygen consumption rates (OCR) were measured under basal conditions and treatment with 20 μM avocatin B and 100 mM etomoxir as positive control.

### FAO and ROS production

FAO was assessed by using the human FAO flow cytometry kit (Abcam, Cambridge, UK). Levels of the FAO enzymes acyl-coenzyme A dehydrogenase very-long chain (ACADVL), acyl-CoA dehydrogenase, C-4 to C-12 straight chain (ACADM), and hydroxyacyl-CoA dehydrogenase/3-ketoacyl-CoA thiolase/enoyl-CoA hydratase trifunctional protein alpha subunit (HADHA) were measured by a FACScan flow cytometer (Becton Dickinson Immunocytometry Systems, San Jose, CA) and analyzed by Cell Quest software (Becton Dickinson).

ROS production was quantified by using the CellROX deep red flow cytometry assay kit which contains SYTOX dead cell stain (Life Technologies, Carlsbad, CA). Flow cytometric data were acquired using a Canto II flow cytometer (BD Biosciences) and analyzed using FlowJo Version 9.5 software (TreeStar, Ashland, OR).

### Fatty acid and glucose uptake

Fatty acid uptake was detected by a fluorometric fatty acid uptake kit (Abcam) and Glucose uptake was measured by Glucose cellular uptake measurement kit (Cosmo Bio, Tokyo, Japan) according to the manufacturers’ protocol.

### mRNA quantification

Total RNA was extracted from cells with the RNeasy Mini Kit (Qiagen, Hilden, Germany). First-strand cDNA was synthesized with oligo(dT) as primer (Superscript II System; Invitrogen, Carlsbad, CA). Real-time reverse-transcriptase PCR (RT-PCR) was performed by the Model 7500 Real-time PCR System (Applied Biosystems, Foster City, CA). Expression of the mRNAs encoding fatty acid binding protein 4 (*FABP4*), carnitine palmitoyltransferase 1 (*CPT1A)*, and *GAPDH* was detected by TaqMan Gene Expression Assays (*FABP4*: Hs01086177_m1; *CPT1A*: Hs00912671_m1; *GAPDH*: Hs99999905_m1; Applied Biosystems). The PCR cycle number that generated the first fluorescence signal above a threshold value (the threshold cycle; C_t_) was determined. The expression of each gene transcript relative to that of *GAPDH* was calculated as follows: relative expression = 100 × 2 exp [−ΔC_t_], where ΔC_t_ is the mean C_t_ of the transcript of interest minus the mean C_t_ of the transcript for *GAPDH*. The C_t_ data from duplicate PCRs were averaged for calculation of relative expression.

### Microarray analysis

Gene expression profiles of the cells were determined by using the Human Gene 1.0 ST Array (Affymetrix, Santa Clara, CA) according to Affymetrix protocols. Signal intensities were measured by using a GeneChip Scanner3000 (Affymetrix) and converted to numerical data by using the GeneChip Operating Software version 1 (Affymetrix). The digitized data were analyzed by GeneSpring 3.2.2 software (Silicon Genetics, Redwood, CA).

### Immunoblot analysis

Immunoblot analysis was performed as previously described^38^. The following antibodies were used: α-tubulin (Sigma-Aldrich, St Louis, MO), LC-3 (MBL, Nagoya, Japan), phosphorylated- (p-)4E-BP1 Thr37/Thr46, 4E-BP1, p-S6 ribosomal protein (S6K) Ser235/Ser236, S6 ribosomal protein, p-AMPKα Thr172, AMPKα, ATF4 and horseradish peroxidase–linked anti-mouse and anti-rabbit IgG (all, Cell Signaling Technology, Danvers, MA).

### Metabolite measurements

Metabolic methanol extracts spiked with an internal standard solution (Human Metabolome Technologies, Inc., Tsuruoka, Japan) were analyzed using a capillary electrophoresis (CE)-connected ESI-time of flight (TOF)-mass spectroscopy (MS) and CE-MS/MS system (CARCINOSCOPE: Human Metabolome Technologies, Inc.) according to the manufacturer’s instructions^39^. Concentrations of metabolites were calculated by normalizing the peak area of each metabolite with respect to the area of the internal standard and using standard curves, which were obtained by three-point calibration.

### Statistical analyses

Differences between groups were assessed by a two-tailed Student *t*-test or a Wilcoxon matched pair test. A *p*-value < 0.05 was considered statistically significant. Unless otherwise indicated, the results are expressed as the mean ± SD of triplicate samples.

## Electronic supplementary material

Supplementary information
