# Supplementary material for: Inhibition of FAO in AML co-cultured with BM adipocytes: mechanisms of survival and chemosensitization to cytarabine
Source: Sci Rep. 2018 Nov 15;8:16837. doi: 10.1038/s41598-018-35198-6 (PMC6237992; doi:10.1038/s41598-018-35198-6)
Supplement: Supplementary file 1 — Supplementary information [file 41598_2018_35198_MOESM1_ESM.docx]

**TITLE**

**Inhibition of FAO in AML co-cultured with BM adipocytes: mechanisms of survival and chemosensitization to cytarabine**

**AUTHORS/AFFILIATIONS**

Yoko Tabe^1,2,3^, Kaori Saitoh^3^, Haeun Yang^3,4^, Kazumasa Sekihara^3,4^, Kotoko Yamatani^3^, Vivian Ruvolo^2^, Hikari Taka^5^, Naoko Kaga^5^, Mika Kikkawa^5^, Hajime Arai^5^, Takashi Miida^3^, Michael Andreeff^2^, Paul A. Spagnuolo^6^, Marina Konopleva^2,*^

Departments of ^1^Next Generation Hematology Laboratory, ^3^Clinical Laboratory Medicine, ^4^Leading Center for the Development Research of Cancer Medicine, and ^5^Division of Proteomics and BioMolecular Science, Juntendo University Graduate School of Medicine, Tokyo, Japan; ^2^Section of Molecular Hematology and Therapy, Department of Leukemia, The University of Texas MD Anderson Cancer Center, Houston, TX, USA; ^6^Departtment of Food Science, University of Guelph, Guelph, Ontario, Canada.

**Corresponding Author:** Marina Konopleva, MD, PhD, Department of Leukemia, The University of Texas MD Anderson Cancer Center, 1515 Holcombe Boulevard, Unit 448, Houston, TX 77030 USA. Phone: 713-792-7261; Fax: 713-563-7355; E-mail: mkonoplev@mdanderson.org

**SUPPROTING INFORMATION**

**
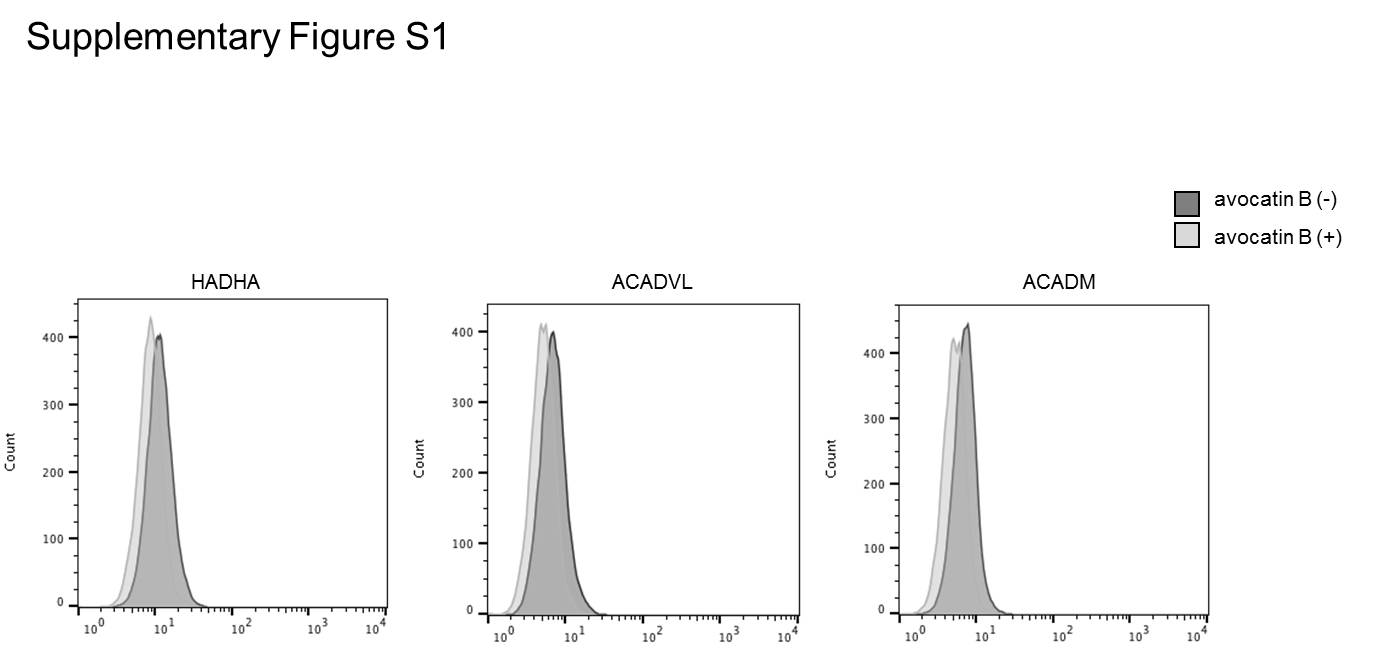
Supplementary Figure S1.**

Avocatin B decreased the levels of FAO cycle enzymes in AML cells co-cultured with BM adipocytes. U937 cells were treated with avocatin B (20 μM) for 24 hours in the presence of BM adipocytes under serum-starved conditions. The significant decrease of the FAO cycle enzymes HADHA, ACADVL and ACADM expression levels after avocatin B treatment was detected by flow cytometry ; % positive cells in control vs avocatin B (+) , HADHA 37.2±4.9 vs 17.1±9.2, p = 0.03, ACADVL 17.6±3.7 vs 11.1±1.1, p = 0.04, ACADM 15.5±2.4 vs 10.1±2.1, p = 0.04). Experiments were performed three times. Representative histograms of levels of HADHA, ACADVL and ACADM are shown.

**Supplementary Figure S2.**

**
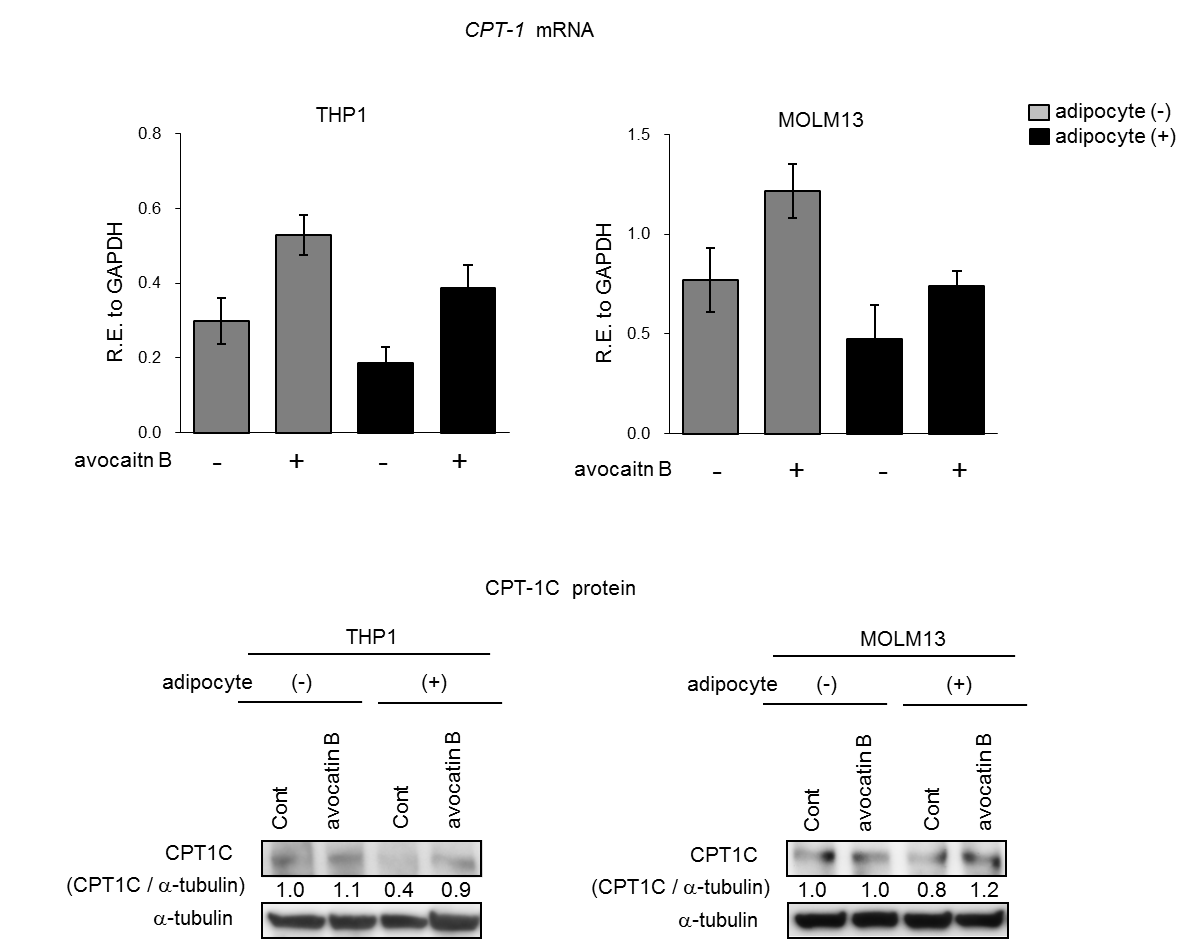
**

THP-1 and MOLM13 cells were cultured with or without avocatin B (10 μM) for 24 hours in the presence or absence of BM adipocytes. *CPT-1* mRNA expression in the cells was determined by quantitative RT-PCR. The expression of transcripts of each gene relative (R.E.) to the expression of GAPDH transcripts was determined as described in Materials and Methods. Graphs show representative data from three independent experiments. CPT1C protein expression levels were detected by immunoblotting; Cont, controls. Results shown are representative of three independent experiments.

**Supplementary Figure S3.**

**
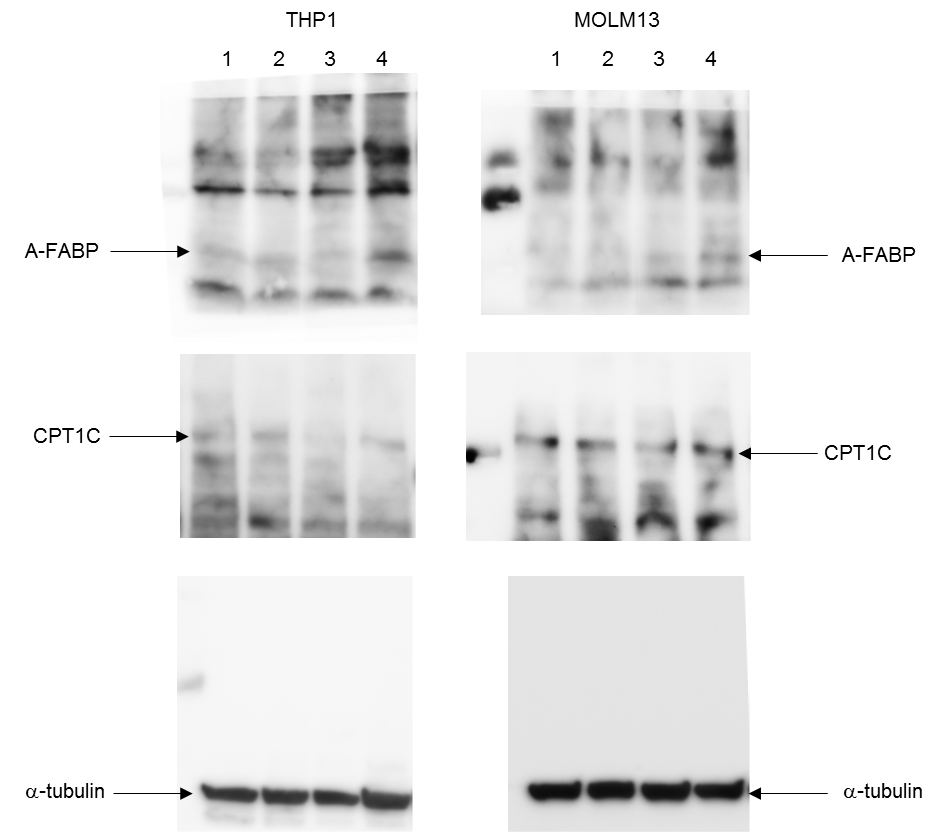
**

THP-1 and MOLM13 cells were co-cultured with BM adipocytes for 24 hours with or without avocatin B (10 μM), and expression levels of A-FABP4 and CPT1C protein were detected by immunoblotting.

Lane 1: control / cultured alone; lane 2: avocatin B treated / cultured alone; lane 3: control / co-cultured with BM adipocytes; lane 4: avocatin B treated / co-cultured with BM adipocytes.

**Supplementary Figure S4.**

**
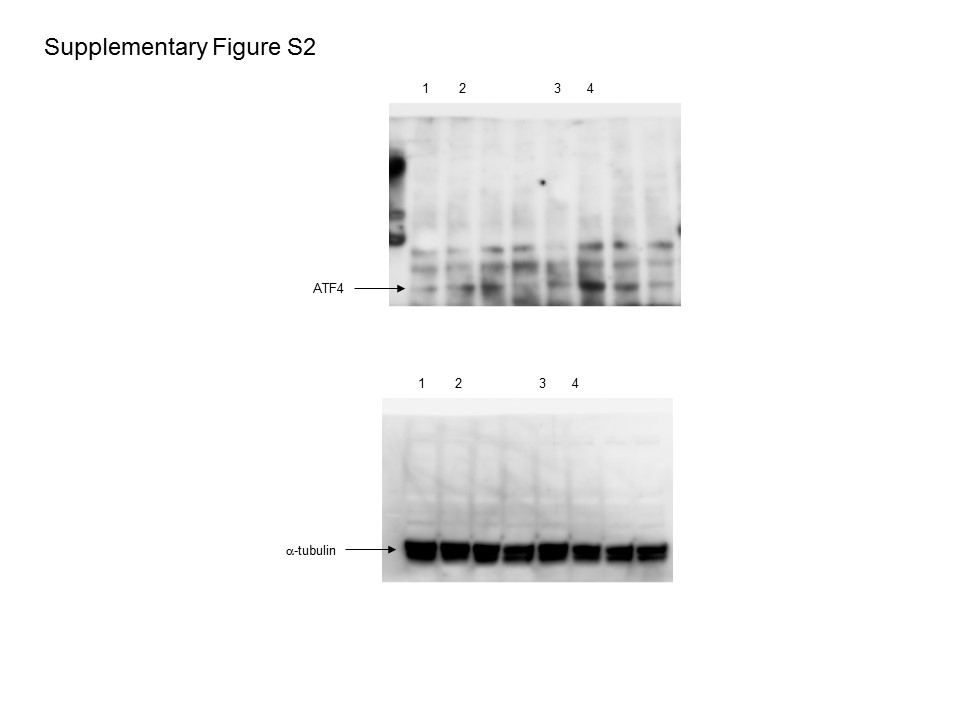
**

THP-1 cells were co-cultured with BM adipocytes for 24 hours with or without avocatin B (10 μM), and expression levels of ATF4 protein were detected by immunoblotting.

Lane 1: control THP-1 cells cultured alone; lane 2: avocatin B treated THP-1 cells cultured alone; lane 3: control THP-1 cells co-cultured with BM adipocytes; lane 4: avocatin B treated THP-1 cells co-cultured with BM adipocytes.

**Supplementary Figure S5.**


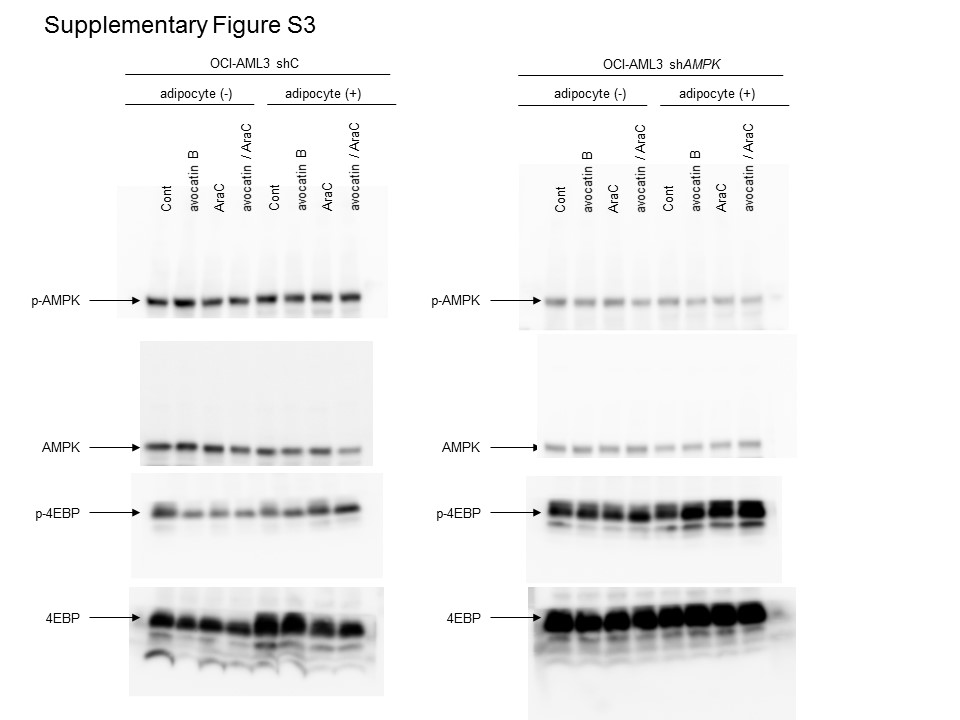


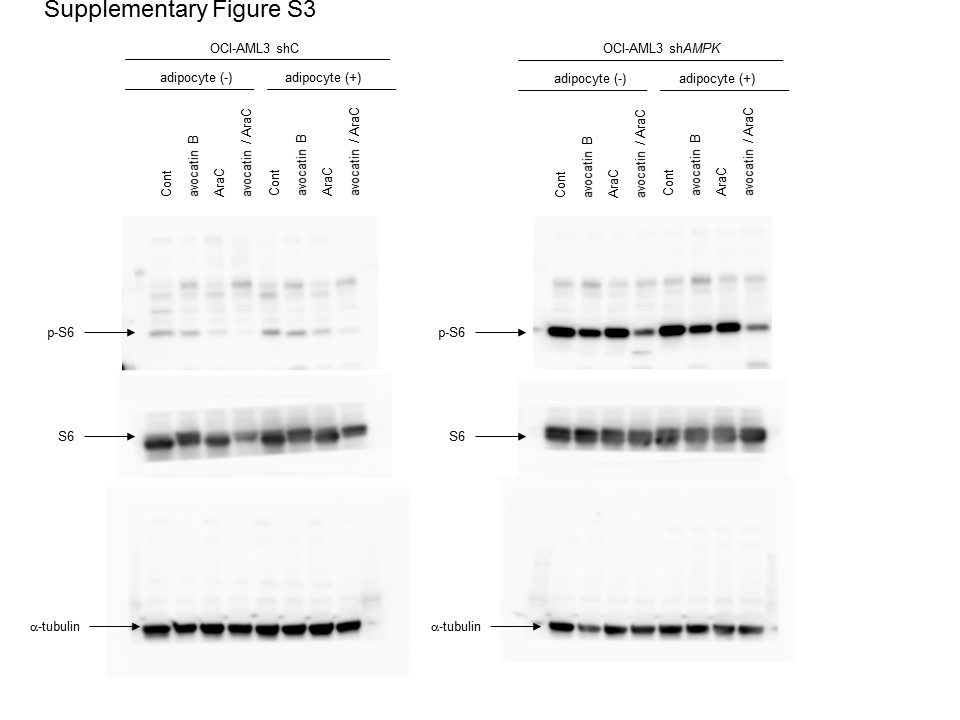


OCI-AML3 cells transfected with control short hairpin RNA (shC) or shRNA against AMPK (shAMPK) were cultured with or without avocatin B (10 μM) and AraC (3 μM) for 18 hours in the presence or absence of BM adipocytes. Expression levels of AMPK, p-AMPK, 4E-BP1, p-4E-BP1, S6, p-S6 and α-tubulin proteins in the cells were detected by immunoblotting; Cont, controls.

**Supplementary Figure S6.**


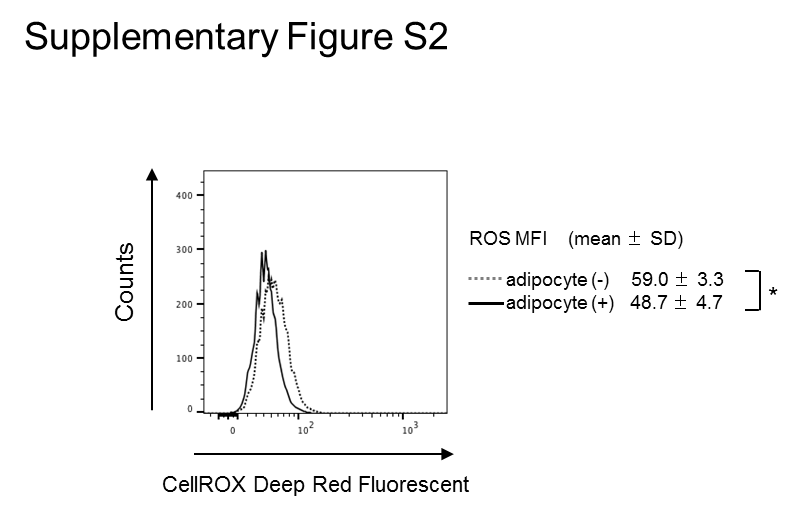


U937 cells were cultured for 24 hours in the presence or absence of BM adipocytes under serum-starved conditions. Representative histogram of CellROX staining (ROS production) in the viable cells (SYTOX staining) under the indicated conditions are shown. Mean fluorescence intensity (MFI) indicates the mean ± SD of results of three independent experiments. *p<0.05.

**
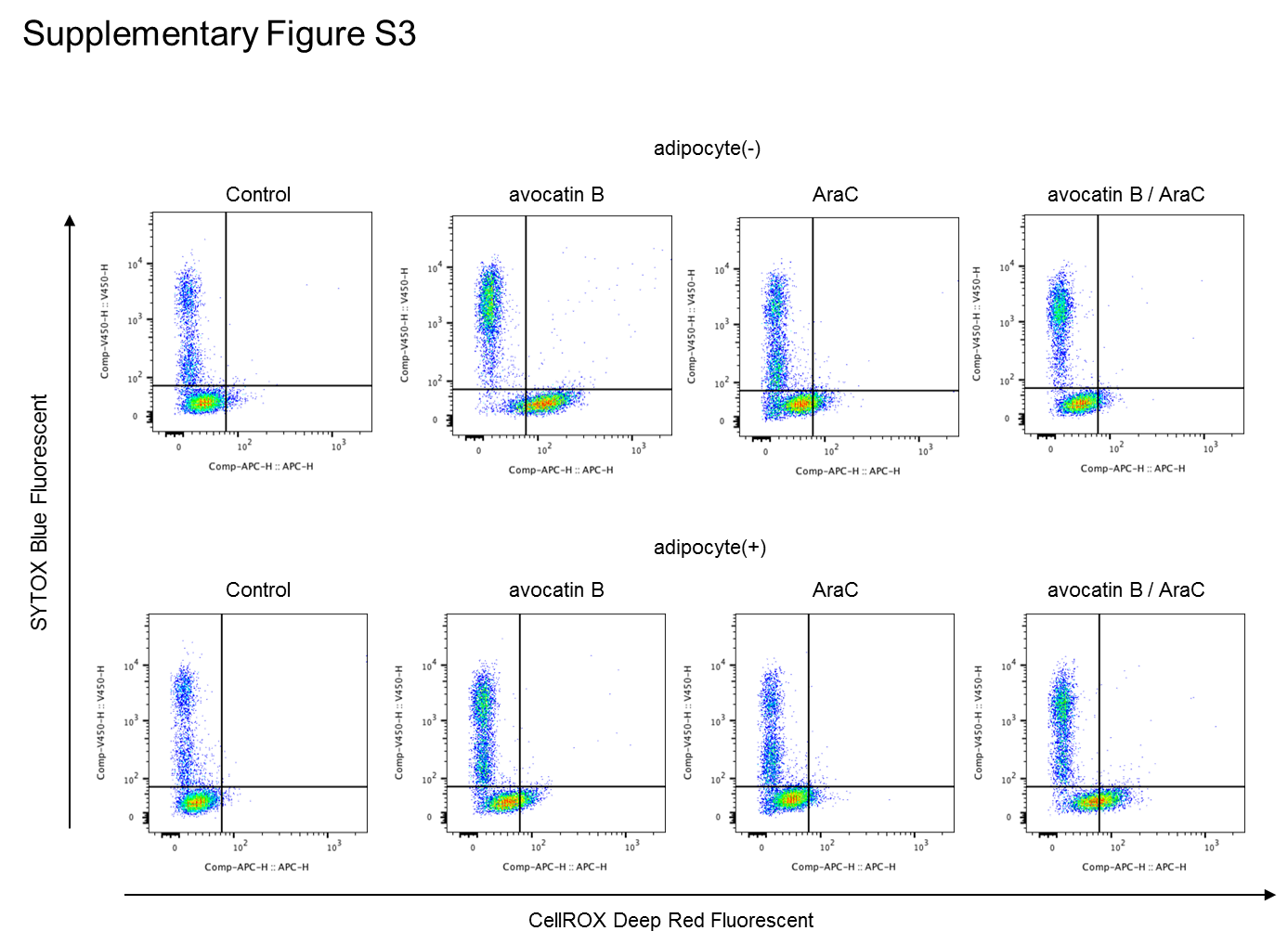
Supplementary Figure S7.**

U937 cells were treated with / without avocatin B (10 μM), AraC (0.1μM) or avocatin B+AraC for 24 hours in the presence or absence of BM adipocytes under serum-starved conditions. Representative flow cytometry plots of CellROX staining (ROS production) and SYTOX staining (dead cells) under the indicated conditions.

**Supplementary Figure S8.**

**
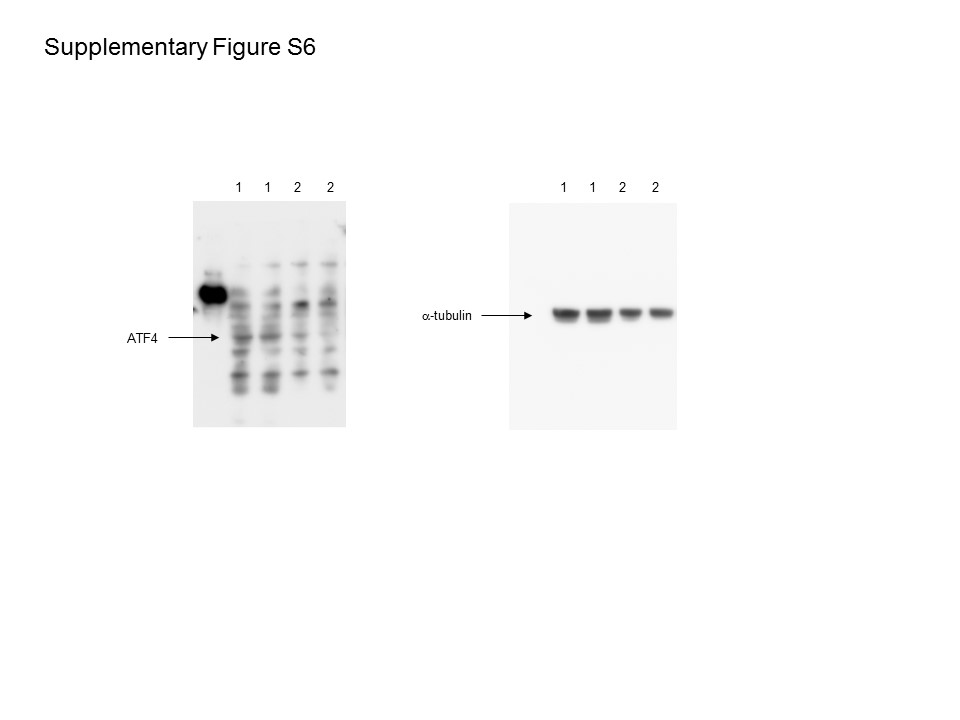
**

OCI-AML3 cells were transfected with control short hairpin RNA (shC) or shRNA against *ATF4* (sh*ATF4*). Immunoblotting were performed on cells using anti-ATF4 antobody or anti-tubulin antibody. Lane 1: control siRNA (shC); lane 2: *ATF4* siRNA (sh*ATF4*).

**Supplementary Figure S9.**

**
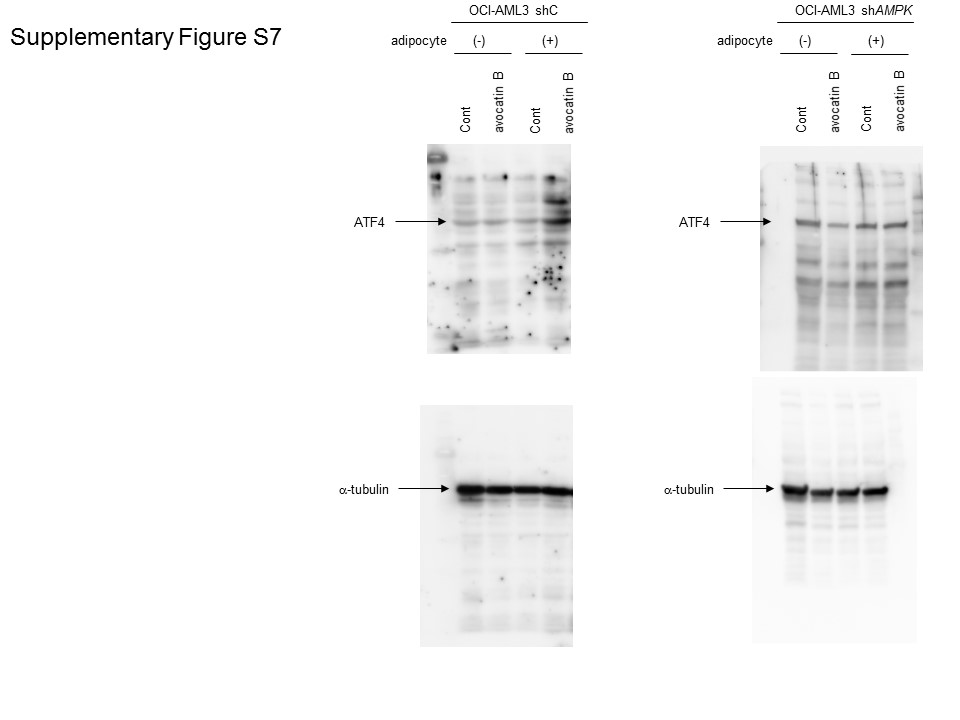
**

OCI-AML3 cells transfected with control short hairpin RNA (shC) or shRNA against *ATF4* (sh*ATF4*) were co-cultured with BM adipocytes for 24 hours with or without avocatin B (10 μM), and expression levels of ATF4 protein were detected by immunoblotting; Cont, controls.

**Supplementary Table S1. IC50 and ED50 of avocatin B in AML cell lines**

**Supplementary Table S2. Quantitative metabolite data in THP-1 cells detected by CE-TOF-MS after BM adipocyte co-culture and avocatin B treatment**

**Supplementary Table S3. Combination indices for avocatin B and AraC in THP1**
